# Acute retinal necrosis presenting exudative retinal detachment: a case report

**DOI:** 10.3389/fmed.2026.1746774

**Published:** 2026-06-24

**Authors:** Han Wang, Ying Zhu, Ai Xuan Cheng, Chao Zhang

**Affiliations:** Department of Ophthalmology, Liaoning Provincial Key Laboratory of Cornea and Ocular Surface Diseases, Liaoning Provincial Optometry Technology Engineering Center, Dalian Third People’s Hospital Affiliated to Dalian University of Technology, Dalian, China

**Keywords:** acute retinal necrosis, antiviral treatment, differential diagnosis, exudative retinal detachment, varicella zoster virus

## Abstract

**Background:**

Acute retinal necrosis (ARN) is a severe, rapidly progressive viral retinitis that is commonly complicated by rhegmatogenous retinal detachment in its late stage. However, the presentation of ARN with exudative retinal detachment (ERD) in the early phase is exceptionally rare, particularly when caused by varicella zoster virus (VZV) in an adult patient. This report highlights this atypical presentation, which initially occurred without definite evidence of retinal necrosis, posing a diagnostic challenge.

**Case presentation:**

A 43-year-old woman presented with acute blurred vision, eye redness, and ocular pain in the left eye of 3 days’ duration. Initial clinical examination revealed ciliary congestion, vitritis, optic disc swelling, and a non-rhegmatogenous retinal detachment. Optical coherence tomography demonstrated optic disc and macular edema with intraretinal cystic spaces and a serous retinal detachment temporal to the fovea. Given the atypical presentation, the patient was initially treated with corticosteroids. Two days later, characteristic peripheral retinal necrotic lesions appeared, prompting immediate aqueous humor sampling. Metagenomic testing confirmed VZV infection. The patient was then treated aggressively with systemic intravenous acyclovir, intravitreal ganciclovir injections, and systemic corticosteroids. This regimen led to rapid resolution of the retinal detachment and complete resolution of the retinal lesions, with stable visual acuity maintained at 1 month of follow-up.

**Conclusion:**

Exudative retinal detachment is a rare manifestation of early-stage ARN. In uveitis patients presenting with ERD who show a poor response to initial anti-inflammatory therapy, viral infection (particularly VZV) should be considered in the differential diagnosis. Aggressive combined systemic and intravitreal antiviral therapy, alongside corticosteroids, is critical for achieving favorable anatomical and visual outcomes in these challenging cases.

## Background

Acute retinal necrosis (ARN) is a rare, vision-threatening panuveitis that can be caused by HSV-1, HSV-2, VZV, and CMV. It is characterized by vascular occlusion, progressive peripheral necrosis, retinitis, and vitritis (1). Rhegmatogenous retinal detachment (RRD) is common in the late stage of ARN because retinal breaks develop along the borders of the necrotic area. The occurrence of retinal detachment (RD) is accompanied by a poor prognosis in ARN patients. However, ARN presenting with exudative retinal detachment (ERD) is rare. We report a rare case of early-stage ARN presenting with ERD.

## Case report

A 43-year-old woman was referred to our hospital with a 3-day history of acute blurred vision in the left eye, accompanied by eye redness and pain with eye movements. No significant medical history was reported except for a mastectomy 20 years ago. She underwent an uncomplicated gastrointestinal polypectomy 1 month before admission. Her family history was non-contributory.

**Figure 1:**
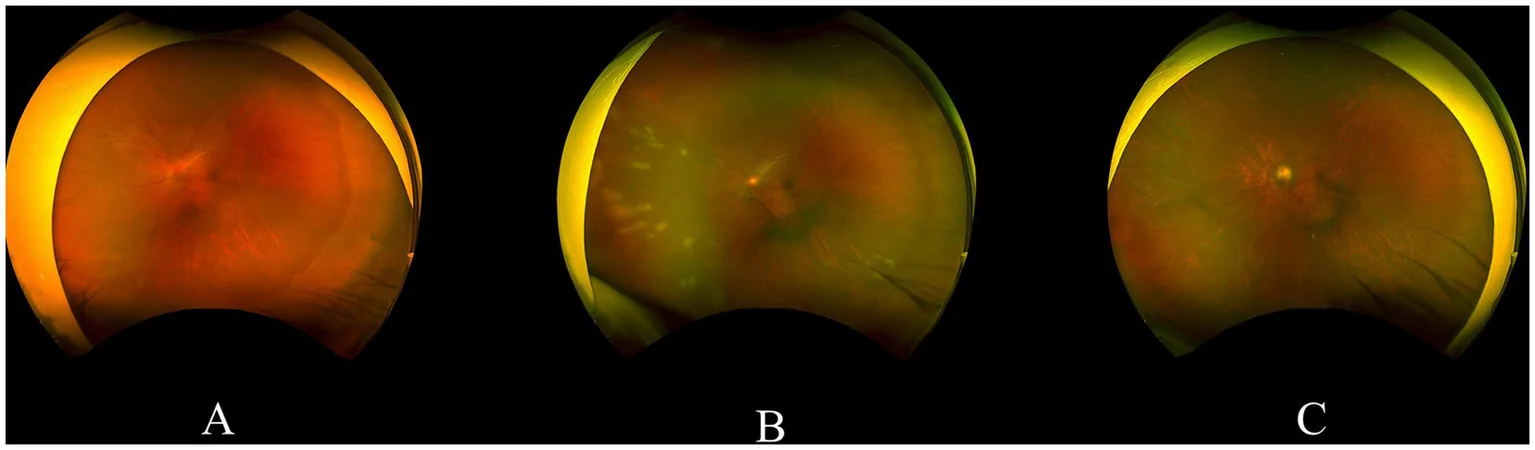
**(A)** Fundus photograph of the left eye on the first visit. **(B)** Fundus photograph of the left eye 2 days later. **(C)** Fundus photograph of the left eye 1 month after treatment.

Clinical examination on admission revealed that the best corrected visual acuity (BCVA), measured using the Snellen chart (decimal scale), was 0.8 in the right eye and 0.1 in the left eye. Ocular pain associated with left eye movement was documented. The bilateral intraocular pressure was 20 mmHg, as measured by non-contact tonometry (Topcon CT-800; Topcon Corporation, Tokyo, Japan). The anterior segment and fundus examinations of the right eye were unremarkable. Slit lamp examination of the left eye revealed ciliary congestion, dust-like keratic precipitates, and 2 + grade cells in the anterior chamber. Fundus examination of the left eye revealed severe vitritis, retinal infiltrates located in the inferior nasal area, and optic disc swelling. The retinal vessels in the inferior nasal quadrant demonstrated tortuous dilation and focal retinal hemorrhages (Figure 1A) Furthermore, detailed fundus examination revealed exudative retinal detachment in the nasal, superior, and temporal quadrants without macular involvement or retinal tears.

**Figure 2:**
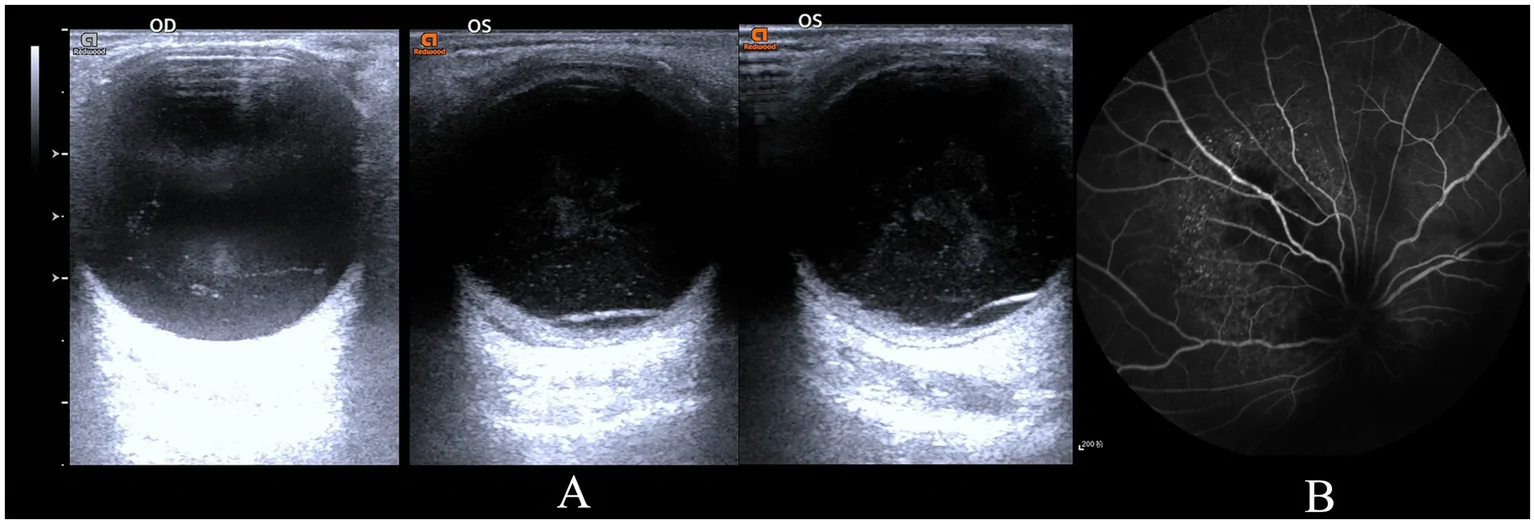
**(A)** B-scan ultrasonography revealed retinal detachment and posterior scleral thickening in the left eye, along with mild vitreous opacities in the right eye. **(B)** Fluorescein angiography of the left eye revealed numerous punctate hyperfluorescent areas superonasal to the optic disc, with subsequent dye leakage.

An ultrasound B-scan revealed retinal detachment and thickening of the posterior sclera of the left eye and mild vitreous opacity of the right eye (Figure 2A).

**Figure 3:**
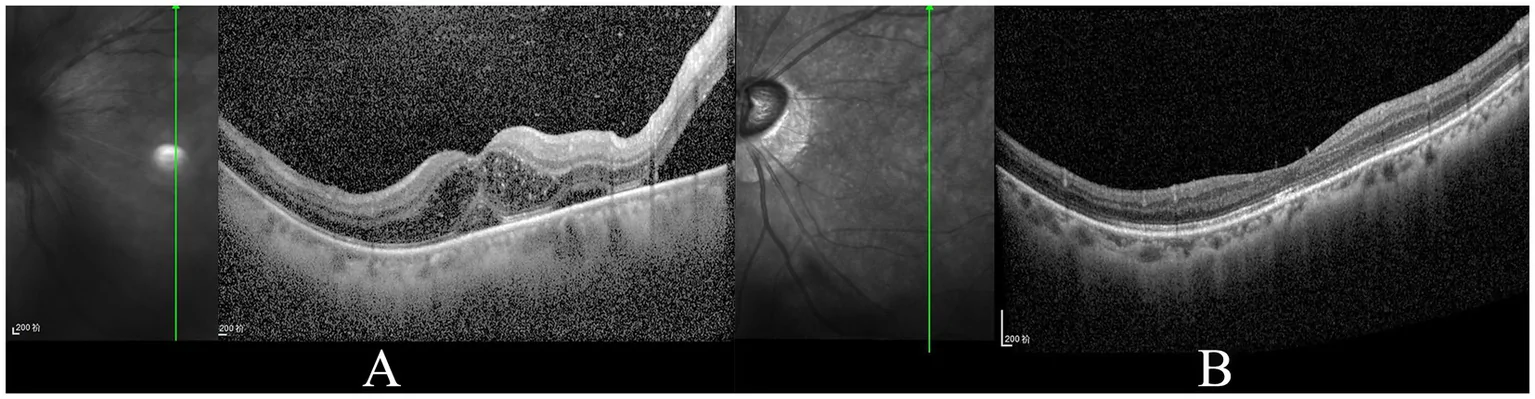
**(A)** Optical coherence tomography (OCT) of the left eye revealed optic disc edema and macular edema, characterized by intraretinal cystic spaces predominantly concentrated within the outer nuclear layer. Numerous associated hyperreflective foci (white dots) were noted in this layer. Additionally, serous retinal detachment was identified temporally from the fovea. **(B)** OCT demonstrated the absence of macular or optic disc edema.

Optical coherence tomography (OCT) of the left eye demonstrated both optic disc and macular edema. The intraretinal cysts were most concentrated in the outer nuclear layer, where many white dots accumulated. Retinal detachment was identified in the temporal region of the fovea. (Figure 3A).

Fluorescein angiography (FA) of the left eye showed localized hypofluorescence in the perimacular region during the early phase. It also revealed numerous punctate areas of hyperfluorescence located superonasal to the optic disc, showing progressive enlargement and increased intensity in the late phase, consistent with dye leakage. (Figure 2B).

There were no remarkable findings in the right eye on fundus photography, OCT, or fluorescein angiography. Laboratory tests, including complete blood count, liver and renal function tests, serum electrolytes, hepatitis serology, syphilis serology, HIV antibody tests, and the T-SPOT. TB assay, revealed no abnormalities. Cranial magnetic resonance imaging (MRI) and chest computed tomography (CT) were unremarkable.

The patient received a posterior periocular injection of dexamethasone (5 mg/1 mL) in the left eye, along with topical 0.1% prednisolone acetate eye drops and 1% atropine sulfate eye gel. After 2 days, while the best-corrected visual acuity of the left eye remained unchanged, ophthalmoscopic examination revealed a degree of improvement in vitreous opacity; meanwhile, multiple necrotic lesions were noted in the peripheral retina, and patchy hemorrhages around the posterior pole of the retina were observed. (Figure 1B) The aqueous humor from the anterior chamber of the left eye was collected immediately for metagenomic testing to rule out infection.

Metagenomic testing yielded positive results for VZV. The diagnosis of ARN was confirmed, and the patient received intravitreal injections of ganciclovir (3 mg/0.1 mL) 2 times per week and systemic antiviral treatment with intravenous acyclovir 800 mg 3 times daily. Three days after the initiation of antiviral therapy, oral prednisone was administered at an initial dose of 1 mg/kg/day, followed by a weekly taper of 10 mg until complete discontinuation.

After 1 week, eye movement pain and visual acuity improved, retinal reattachment was achieved, and the size and number of active retinitis foci decreased. After 2 weeks, the retinal lesions resolved, with the left eye maintaining stable visual acuity. OCT demonstrated the complete resolution of both macular and optic disc edema, showing a restored retinal profile (Figure 3B). We collected the aqueous humor from the anterior chamber, and the PCR result for VZV was 5,690 copies/mL. Antiviral treatment was switched from intravenous acyclovir to oral famciclovir 500 mg three times daily, and the frequency of intravitreal ganciclovir injections was reduced to once weekly. One month post-intervention, VZV DNA PCR analysis of the aqueous humor revealed no VZV copies, and no retinal lesions were observed. (Figure 1C), with the left eye maintaining stable visual acuity. The patient was advised to continue oral famciclovir (500 mg, three times daily) for 3 months as part of the long-term antiviral regimen.

## Discussion

We report a case of exudative retinal detachment as a rare manifestation of the early stage of ARN. Owing to the atypical presentation of retinal infiltrates combined with exudative retinal detachment, we were unable to diagnose acute retinal necrosis at initial presentation. Diagnosing ARN can be challenging because of its variable clinical presentation and severity. A comprehensive assessment of the patient’s previous medical history, prodromal symptoms, clinical characteristics, and laboratory investigations is vital for establishing the diagnosis of ARN. Given the initial clinical presentation—characterized by intense vitritis and whitish retinal lesions—the differential diagnosis primarily includes Behçet disease and infectious endophthalmitis. Behçet disease was a significant consideration due to the presence of severe intraocular inflammation. This entity is typically distinguished from ARN by its systemic involvement, such as recurrent oral and genital aphthous ulcers and erythema nodosum. Crucially, Behçet disease lacks the discrete, rapidly expanding, and confluent peripheral retinal necrosis that defines the viral pathology of ARN. Infectious endophthalmitis, a medical emergency, was also considered due to the patient’s acute ocular pain and diffuse vitreous opacification. However, endophthalmitis is usually associated with a history of recent trauma or intraocular surgery, or a source of systemic infection, neither of which was present in this patient. Although the left eye presented with significant posterior scleral thickening and ocular pain with extraocular movements, no classic “T-sign” (fluid in Tenon’s space around the optic nerve) was observed. This suggests that the scleral involvement was a reactive secondary inflammation change induced by intense viral replication and adjacent tissue destruction, rather than primary posterior scleritis.

Viral serologies are not the standard for definitive ARN diagnosis. Aqueous humor and vitreous biopsies remain the gold standard for the diagnosis of many infectious uveitides and are particularly valuable when a viral etiology is suspected. We diagnosed ARN when necrotic lesions occurred, and PCR of the aqueous humor confirmed VZV infection. Detailed fundus examination ruled out retinal tears and tractional vitreoretinopathy. The retinal detachment resolved following antiviral and corticosteroid therapy, supporting the diagnosis of exudative retinal detachment. Additionally, mild vitreous opacities were observed in the patient’s right eye at presentation. Given that the right eye maintained stable visual acuity and showed no evidence of active retinitis, retinal vasculitis, or optic disc swelling on multimodal imaging throughout the entire follow-up period, these opacities were clinically interpreted as benign, physiological vitreous degeneration.

ARN is a devastating form of viral retinitis, the early stage of which is characterized by rapid-onset peripheral necrotizing retinitis, occlusive vasculopathy, and intense intraocular inflammation. VZV is the most common causative agent of acute retinal necrosis, whereas HSV is more frequently identified in younger patients (2). CMV-associated retinal necrosis is mainly observed in immunocompromised patients. The disease progresses rapidly without prompt diagnosis or effective treatment. RRD is commonly complicated by proliferative vitreoretinopathy (PVR) and affects up to 39–61.1% of ARN patients; it most often occurs during the first 3 months (3, 4). Manifestation of ERD in patients with early-stage ARN is rare. Cases of unilateral HSV-1-associated ARN presenting with ERD have been reported by Patel (5) and Duker (6). Furthermore, five cases of ERD with HSV-associated ARN infection have been reported in children (2, 7–9). Although cases of VZV-related ARN complicated by ERD have been reported, this association remains exceptionally rare, with very limited documentation in the literature. Adamska et al. (10) reported a case of a 56-year-old immunocompetent man who presented with ERD; however, in that specific case, the detachment occurred subsequent to the definitive appearance of peripheral retinal necrosis. In contrast, our patient presented with ERD at the very early stage, prior to the clinical onset of visible retinal necrosis. This highlights the novelty of the present case and underscores the importance of recognizing ERD as a potential initial or prodromal manifestation of ARN.

The pathogenesis of ERD fundamentally arises from a disruption in the equilibrium between subretinal fluid production and RPE-mediated absorption. In inflammatory ocular diseases, excessive fluid leakage from the choroid can exceed the physiological clearance capacity of the RPE, leading to fluid accumulation and overwhelming exudation into the subretinal space (11). This mechanism is highly consistent with our multi-modal imaging, which demonstrated pronounced uveal inflammation and a serous detachment that responded favorably to timely combination therapy. Current treatments for ARN include early initiation of systemic antiviral therapy combined with intravitreal injections of antiviral agents and adjunctive systemic corticosteroids. Current clinical consensus among retinal experts suggests that systemic corticosteroids should be initiated 24–48 h after the initiation of antiviral therapy to mitigate inflammatory complications (12). Initiating corticosteroid therapy without antiviral prophylaxis can trigger severe, treatment-resistant ARN, significantly compromising the patient’s long-term visual prognosis (12). Due to delayed diagnosis and inadequate combined systemic and intravitreal antiviral therapy, the condition of ARN patients from Duke and Adamska progressed rapidly, resulting in a relatively poor prognosis (6, 10). Through early diagnosis and aggressive management involving systemic antiviral and corticosteroid agents, intravitreal ganciclovir injections, and sustained oral antiviral maintenance therapy during follow-up, the condition of our patient was effectively controlled, resulting in satisfactory visual outcomes.

Several limitations of this study should be acknowledged. First, due to the atypical initial clinical presentation, metagenomic next-generation sequencing (mNGS) was used rather than targeted pathogen-specific assays, thereby increasing overall diagnostic costs. Second, the follow-up period was limited to 1 month. This duration is relatively short to fully evaluate long-term outcomes; therefore, continued monitoring of the patient’s prognosis is planned.

Our results indicate that ERD can occur at the early stage in patients with ARN. If a uveitis patient with ERD shows a poor response to conventional anti-inflammatory therapy, the possibility of viral infection should also be considered. Additionally, combined systemic and intravitreal ganciclovir therapy, along with systemic corticosteroids, is safe and effective for early-stage ARN.

## Data Availability

The raw data supporting the conclusions of this article will be made available by the authors, without undue reservation.
