# Correction: Acute retinal necrosis presenting exudative retinal detachment: a case report

**DOI:** 10.3389/fmed.2026.1947701

**Published:** 2026-08-14

**Authors:** Han Wang, Ying Zhu, Ai Xuan Cheng, Chao Zhang

**Affiliations:** Department of Ophthalmology, Liaoning Provincial Key Laboratory of Cornea and Ocular Surface Diseases, Liaoning Provincial Optometry Technology Engineering Center, Dalian Third People's Hospital Affiliated to Dalian University of Technology, Dalian, China

**Keywords:** acute retinal necrosis, antiviral treatment, differential diagnosis, exudative retinal detachment, varicella zoster virus

Author Ying Zhu was erroneously omitted as equal contributing author. The equal **contribution statement** “These authors have contributed equally to this work and share first authorship” has now been added to author Han Wang and author Ying Zhu.

The original version of this article has been updated.

